# Supplementary material for: Quantum barriers engineering toward radiative and stable perovskite photovoltaic devices
Source: Nat Commun. 2024 May 28;15:4547. doi: 10.1038/s41467-024-48887-w (PMC11133308; doi:10.1038/s41467-024-48887-w)
Supplement: Supplementary file 1 — Supplementary Information [file 41467_2024_48887_MOESM1_ESM.pdf]

Supplementary Information

Quantum Barriers Engineering toward Radiative and Stable Perovskite Photovoltaic Devices

Kyung Mun Yeom, Changsoon Cho, Eui Hyuk Jung, Geunjin Kim, Chan Su Moon, So Yeon Park, Su Hyun Kim, Mun Young Woo, Mohammed Nabaz Taher Khayyat, Wanhee Lee, Nam Joong Jeon, Miguel Anaya, Samuel D. Stranks, Richard H. Friend, Neil C. Greenham, Jun Hong Noh

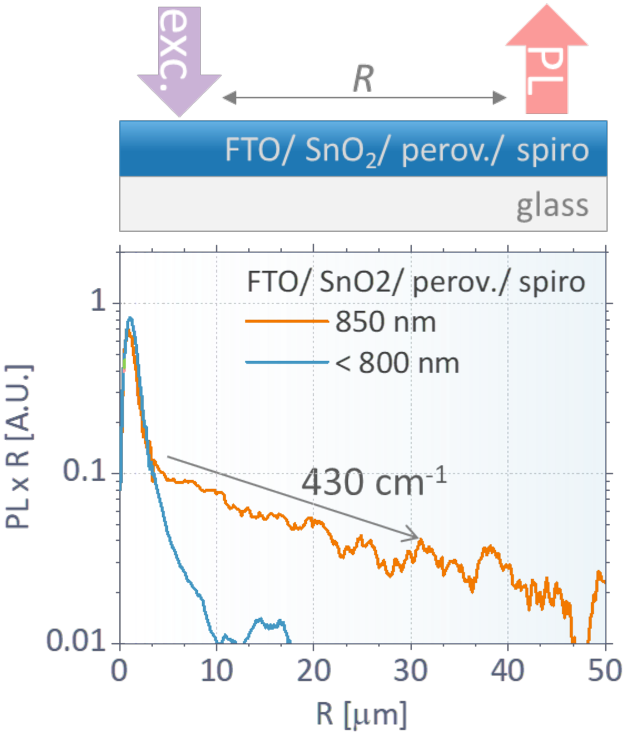

Supplementary Figure 1. Spatially resolved PL profile on the structure of glass / FTO / SnO_2 / perovskite (3D/C18) / spiro-OMeTAD, varying the distance (*R*) between confocal excitation and collection spots [1-3]. The profile measured at a long wavelength (850 nm, with a Thorlabs FB850-40 bandpass filter) exhibits a decaying tail representing the scattering loss, whereas that at a short wavelength (<800 nm, with a Thorlabs FES0800 shortpass filter) decays significantly faster owing to the large absorption coefficient of perovskite [2,3].

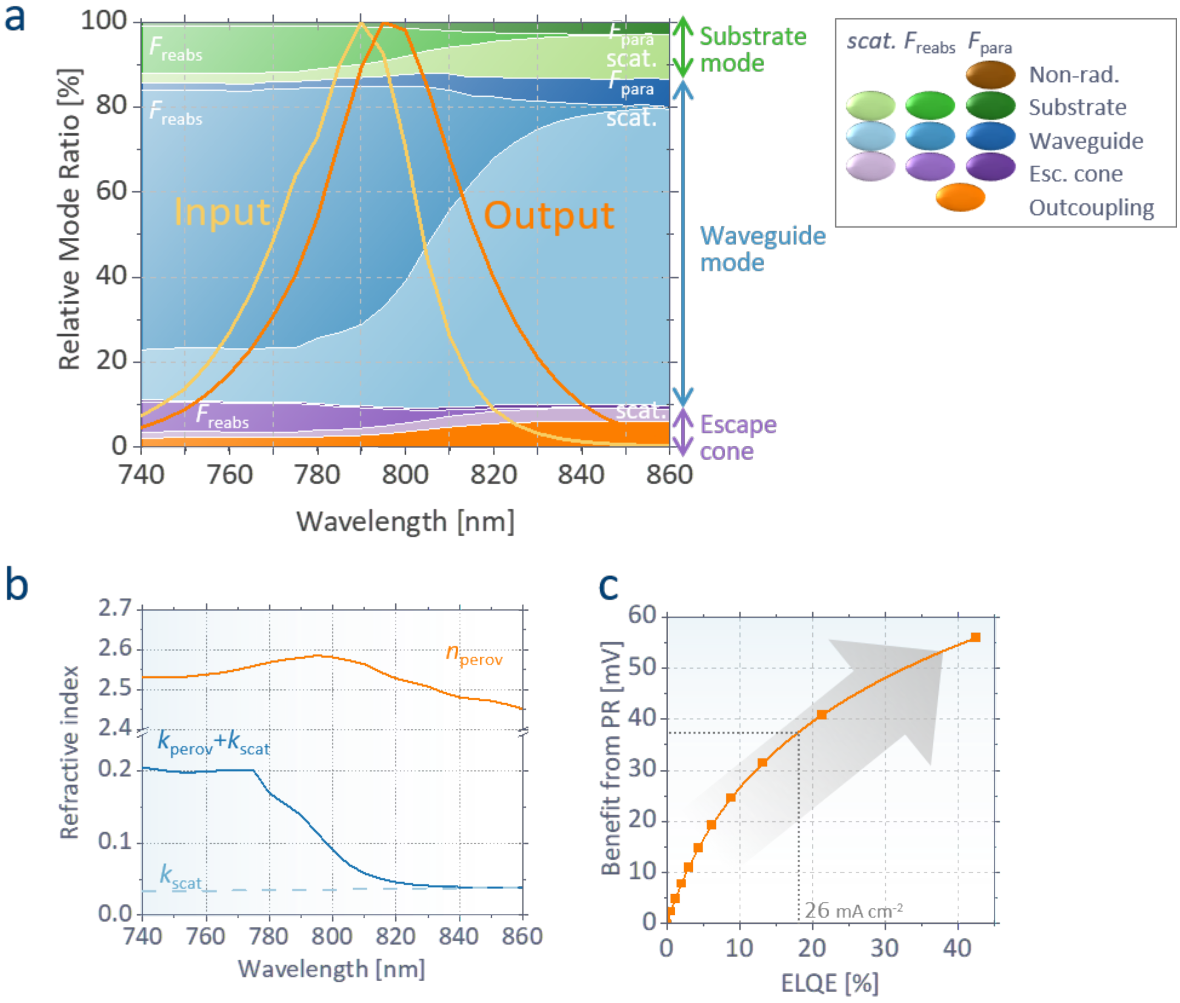

Supplementary Figure 2. **a,** Calculated fractions of various optical propagation modes of photons radiated in the proposed perovskite photovoltaic device (PPV), determined by the propagation angle profiles at each wavelength. Non-outcoupled photons at each mode are eventually reabsorbed by perovskite (F_{reabs}), lost through parasitic absorption (F_{para}), or scattered. The output electroluminescence (EL) spectrum is obtained from the measured spectrum, while the input is reversely calculated from the output, considering the spectral effects of direct outcoupling, scattering, and photon recycling. **b,** Refractive indices used for the calculation. n_{perov} and k_{perov} are obtained using an ellipsometer, while k_{scat} is obtained from the spatially resolved photoluminescence (PL). **c,** Calculated voltage benefit of photon recycling, based on the electroluminescence quantum efficiencies (ELQEs) in Figure 1b.

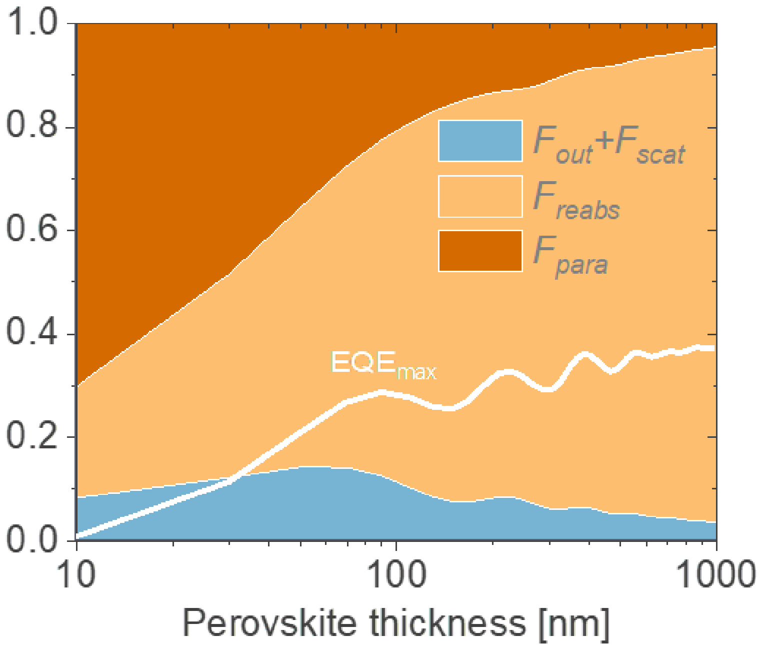

Supplementary Figure 3. Calculated fractions of F_{out} + F_{scat}, F_{reabs}, and F_{para}, as a function of perovskite thickness in our PPV structure, exhibiting the increased perovskite reabsorption and decreased parasitic absorption (hence, increased maximum electroluminescence efficiency (EQE_{max})) in the devices with thicker perovskites.

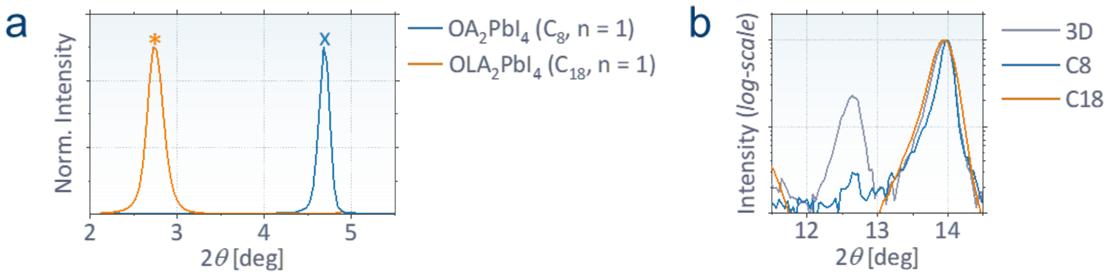

Supplementary Figure 4. a, X-ray diffraction (XRD) analysis of two-dimensional (2D) perovskite films obtained by spin-coating (3,000 rpm, 30 s) of solutions containing 2M octylammonium iodide (or oleylammonium iodide) and 1M PbI_2 (in DMF) directly on the fluorine-doped tin oxide substrates. All films are annealed at 150 °C for 10 min. The OA_2PbI_4 (n = 1) and OLA_2PbI_4 (n = 1) films have peaks at 4.69° and 2.74°, corresponding to the lattice parameters of 1.9 and 3.2 nm, respectively. The peaks for the 3D/C8 (3.56°; 2.5 nm) and 3D/C18 (2.33°; 3.8 nm) demonstrate that the proposed 2D perovskites on the 3D layer possess 0.6 nm larger lattice parameters than OA_2PbI_4 and OLA_2PbI_4 with n = 1, respectively, indicating the formation of n = 2 crystal structures. **b,** XRD of 3D perovskite films with and without MQWs at larger angles, having peaks at 12.7° (PbI_2) and 14.0° (3D perovskite), respectively. The peak for PbI_2 is reduced for the films with organic treatments as PbI_2 reacts with them and forms 2D perovskites.

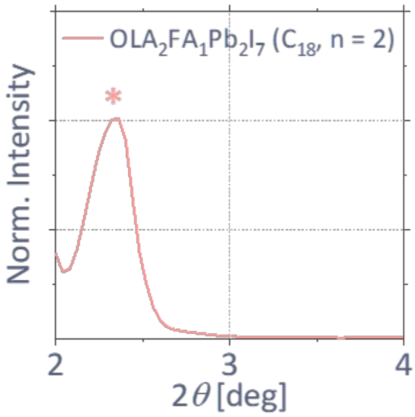

Supplementary Figure 5. X-ray diffraction (XRD) analysis of two-dimensional (2D) perovskite films obtained by spin-coating (3,000 rpm, 30 s) of solutions containing 2 M oleylammonium iodide (OLAI), 1 M formamidinium iodide (FAI), and 2 M PbI_2 (in DMF) directly on the fluorine-doped tin oxide substrates. All films are annealed at 150 °C for 10 min. The $OLA_2FAPb_2I_7$ (*n* = 2) has a peak at 2.33°, consistent with the result for 3D/C18 in Figure 2b.

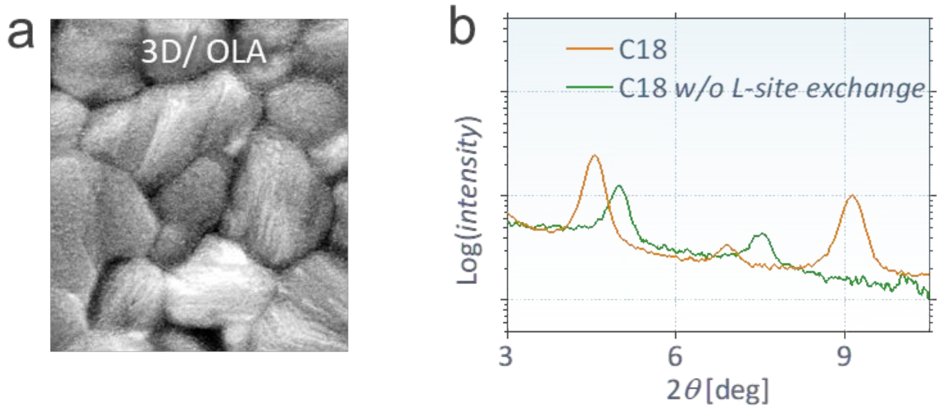

Supplementary Figure 6. a, Scanning electron microscope (SEM) image and **b,** X-ray diffraction (XRD) patterns of C18 2D perovskites without the L-site exchange process (i.e. Oleylamine directly coated on 3D perovskites). The L-site exchange process is shown to shift the XRD peaks to the smaller angles, indicating the increased octahedral number.

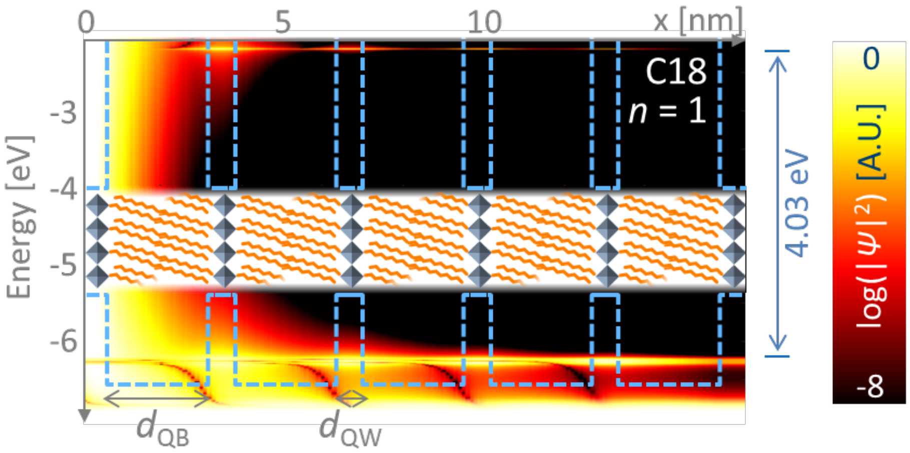

Supplementary Figure 7. Calculated probability density ($|\Psi(x)|^2$, per nm) of electrons (> –4.0 eV) and holes (< –5.4 eV) at each energy level injected from $x = 0$ in 5-stacked QWs with C18 barriers consisting of a thinner d_{QW} of 0.6 nm, corresponding to the $n = 1$ octahedral sheet, exhibiting a large bandgap.

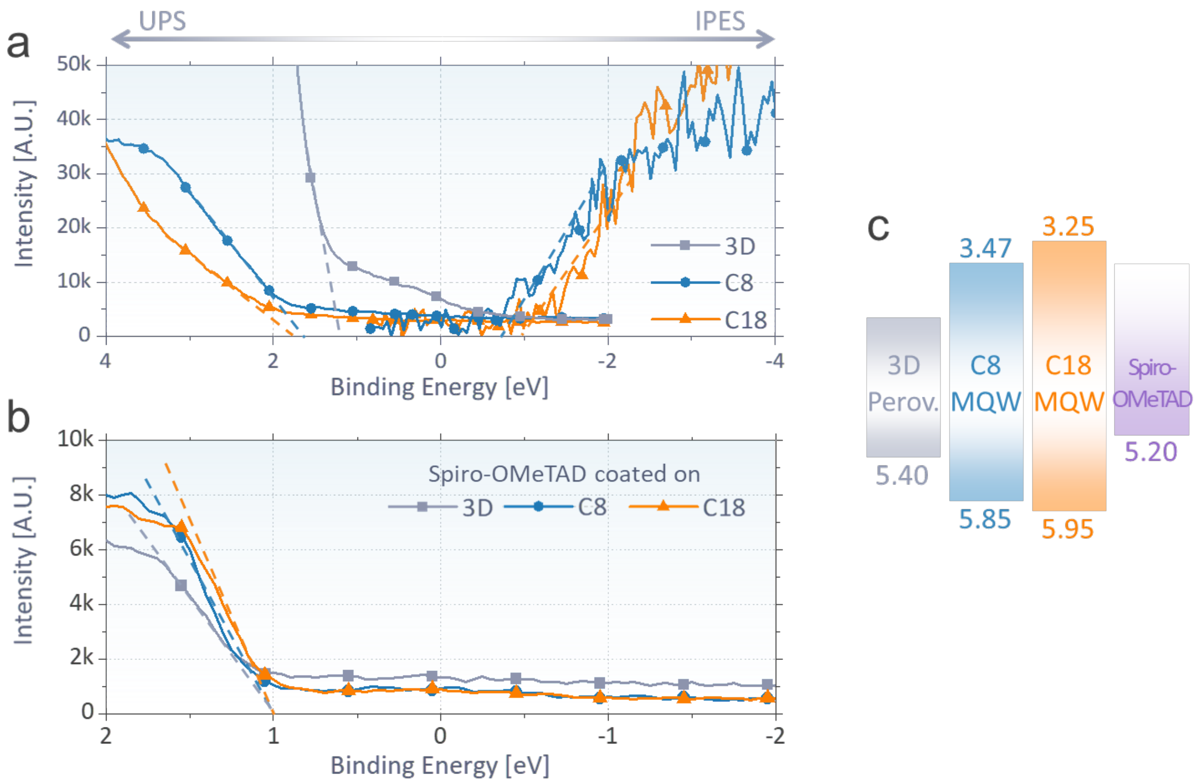

Supplementary Figure 8. a, The surface energy levels of our 3D perovskites with and without C8 or C18 multiple quantum wells, characterized by ultraviolet photoelectron spectroscopy (UPS) and inverse photoelectron spectroscopy (IPES). **b,** Those of our spiro-OMeTAD layers coated on perovskites. **c,** The energy levels (in eV) quantified by fitting the asymptote lines and assuming the valence band of 3D perovskites to be 5.40 eV.

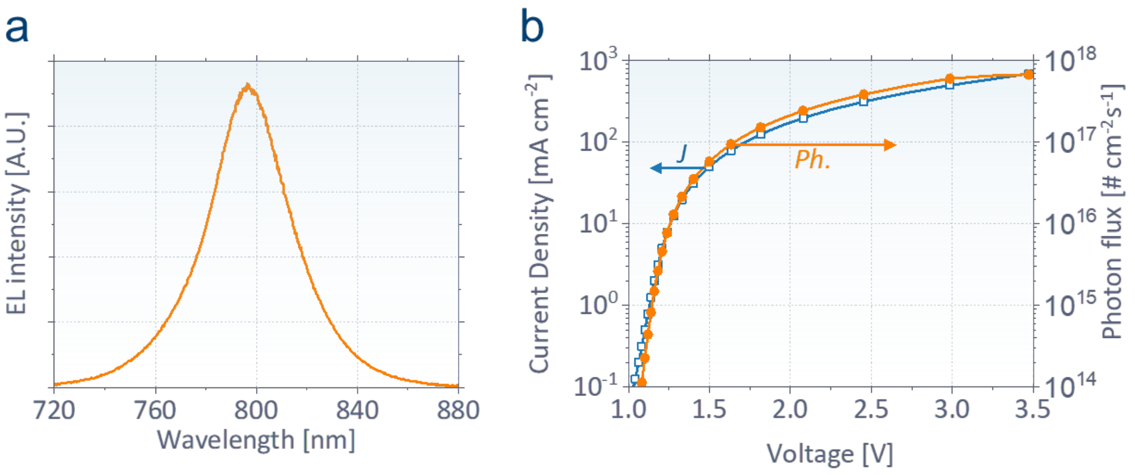

Supplementary Figure 9. a, EL spectrum at the injection of 26 mA cm^{-1} and **b,** dark *J*-*V* curve and output photon flux (= $\eta_{EL} \times J/q$, $q = 1.6 \times 10^{-19}$ C) measured in our brightest device with C18.

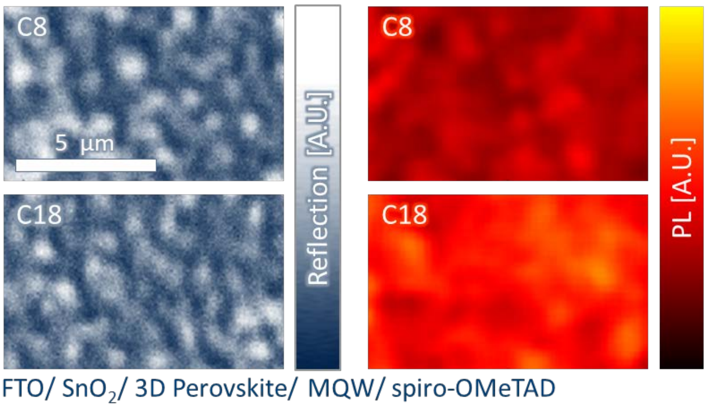

Supplementary Figure 10. Confocal maps of reflectance and PL intensity (at the same areas) for full device structures excluding metal electrodes. While PPVs with C8 exhibit a grain-to-grain difference in PL intensity, those with C18 improve the PL intensity uniformly over the whole domain, confirming their optical benefits.

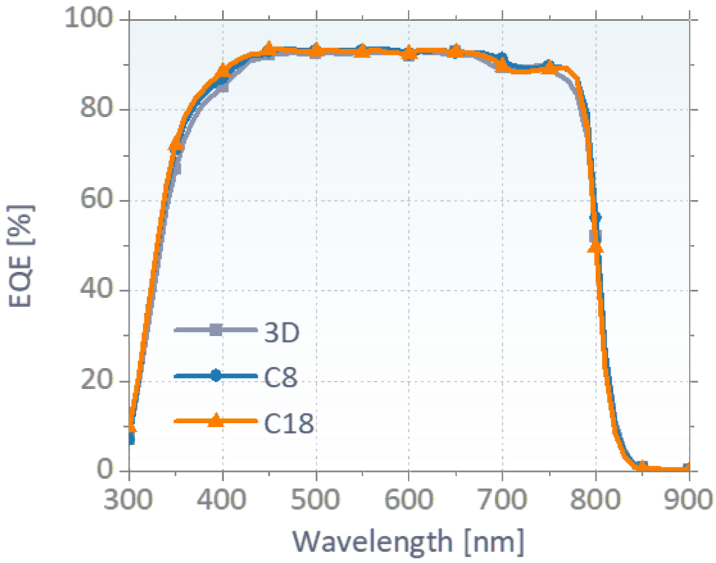

Supplementary Figure 11. EQE spectra of PPVs without and with C8 and C18 measured at Newport.

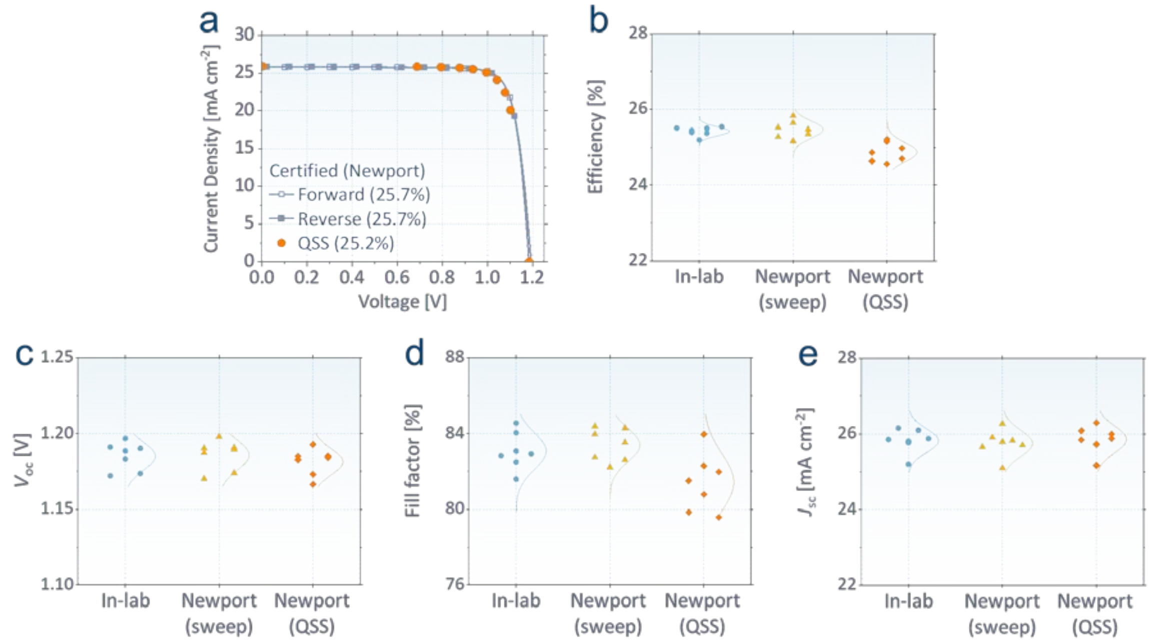

Supplementary Figure 12. a, Electrical performances in forward, reverse, and quasi-steady state (QSS) scans under 1-sun AM1.5G illumination of our PPV with C18, certified by Newport. **b-e,** The efficiency, open-circuit voltage (V_{oc}), fill factor (*FF*), and short-circuit current density (J_{sc}) of the proposed PPVs measured in our laboratory (average sweep) or Newport (average sweep or QSS). The efficiency drop mostly occurs from the *FFs* reduced from 83.4% (average sweep) to 81.4% (QSS) in average in Newport. All devices were measured without encapsulation.

mks Newport™

ACCREDITED Calibration Cert. # 2893.01

Technology and Application Center
PV Lab

Newport Calibration Cert. # 2498

DUT S/N: 7225356 D1-P4
Newport Calibration #: 2498
Manufacturer: Korea University Advanced Energy Conversion Materials & Devices Lab
Material (single junction): Perovskite
Measurement Date: 30-Apr-2020
Temperature Sensor: TC-K, DUT Temperature: 24.6 ± 0.7 °C
Environmental conditions at the time of calibration: Temperature: 24 ± 3 °C; Humidity: 30 ± 20 %

The above DUT has been tested using the following methods to meet the ISO 17025 Standard by the PV Lab at Newport Corporation. Quoted uncertainties are expanded using a coverage factor of $k = 2$ and expressed with an approximately 95% level of confidence. Measurement of total irradiance is traceable to the World Radiometric Reference (WRR) and all other measurements and uncertainties are traceable to NIST and the International System of Units (SI).

***Designated area as defined by thin opaque aperture mask.**

†Reported performance parameters were measured under a 10-point IV sweep configuration wherein the bias voltage (current for Voc determination) is held constant until the measured current (voltage for Voc) is determined to be unchanging at the 0.07% level. This is intended to represent the quasi-steady-state performance of the device. Total IV measurement time under these conditions was approximately 9 minutes.

Efficiency [%]	25.16 ± 0.80	V_oc [V]	1.1851 ± 0.0091	I_sc [A]	0.002477 ± 0.000052
P_max [mW]	2.407 ± 0.071	V_max [V]	1.030 ± 0.015	I_max [A]	0.002338 ± 0.000053
FF [%]	82.0 ± 1.6	Area [cm^2]	0.09570 ± 0.00070	M	1.020 ± 0.015

Methods:

I-V: ASTM E948-16 *Standard Test Method for Electrical Performance of Photovoltaic Cells Using Reference Cells Under Simulated Sunlight*

QE: ASTM E1021-15 *Standard Test Method for Spectral Responsivity Measurements of Photovoltaic Devices*

Standard Reporting Conditions:

Spectrum: AM1.5-G (ASTM G173-03/IEC 60904-3 ed. 2)
1000.0 W/m^2 at 25.0°C

Secondary Reference Cell:

Device S/N: PVM 746
Device Material: GaAs
Window Material: BK7
Certification: National Renewable Energy Laboratory
A2LA accreditation certificate # 2236.01
ISO Tracking #: 2001
Certified short circuit current (I_{sc}) under standard reporting conditions (SRC): 106.02 mA
Calibration due date: 23-Apr-21

Solar Simulator:

Spectrum: Newport Corporation filename *Sol3A_Spectroradiometer_Scan_0215.xls*
Total irradiance: 1000 W/m^2 based on I_{sc} of the above Secondary Reference Cell

Quantum Efficiency for DUT:

Newport Corporation filename *QE 7225356, D1-P4, 0,92 mA WLB.log*
Spectral mismatch correction factor: M = 1.020 ± 0.015

DUT Calibration Procedures:

Newport Corporation document WI1 (EQE).docx
Newport Corporation document Area Measurement WI2 (Area).docx
Newport Corporation document WI3 (IV.Sweep).docx

Cal Cert V1.8	Issue Date: Jun 16, 2021	Page 2 of 2
	Reviewed and Approved by: Geoffrey Wicks (Geoffrey.Wicks@mksinst.com)	
	This certificate to be reproduced in part only with written permission from the Newport PV Laboratory	

Supplementary Figure 13. Certification of the device efficiency from accredited photovoltaic test laboratory (Newport, USA). This certificate is with written permission from Newport.

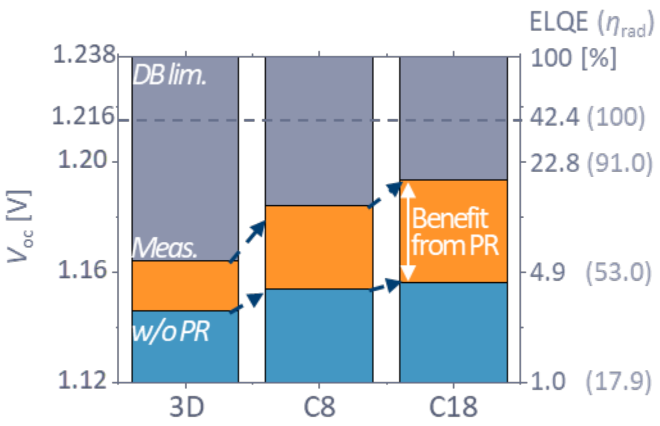

Supplementary Figure 14. V_{oc} of PPVs with and without C8 and C18 MQWs, including and excluding the benefit of photon recycling, respectively. The dashed line represents a practical voltage limit of 1.216 V for an ELQE of 42.4 %, corresponding to the maximum external radiation efficiency calculated for the present PPV architecture with an η_{rad} of 100 %.

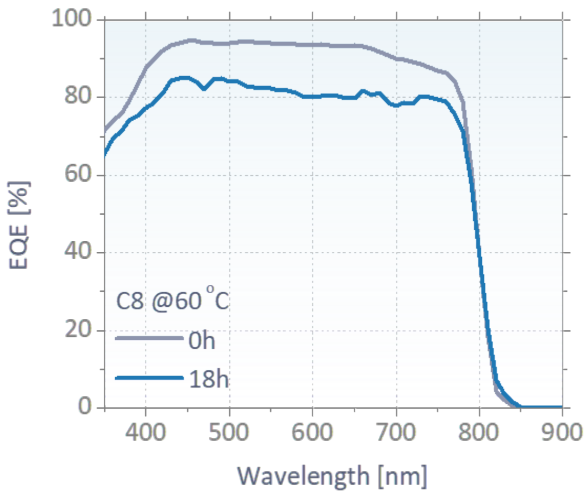

Supplementary Figure 15. EQE spectra of our C8 control devices before and after storage at 60 °C in air without encapsulation. EQE decreases uniformly over the whole spectral range, implying that the decrease in photocurrent density (Figure 3e) mainly comes from the electrical loss (i.e. charge transport) rather than a change in their optical properties.

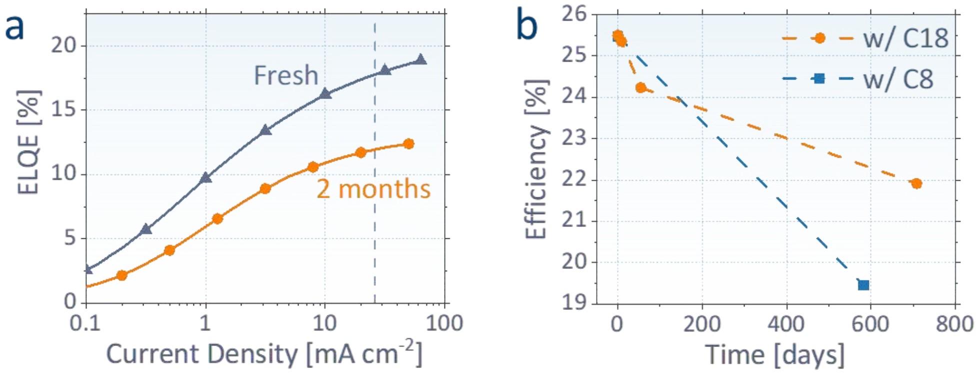

Supplementary Figure 16. The long-term stability of PPVs stored in air at room temperature without encapsulation. **a,** ELQEs of our PPV with C18, where the $\eta_{EL}(J_{ph})$ changes from 17.8% to 11.9% in two months. **b,** Photovoltaic efficiency changes of our PPVs with C8 and C18 in approximately two years.

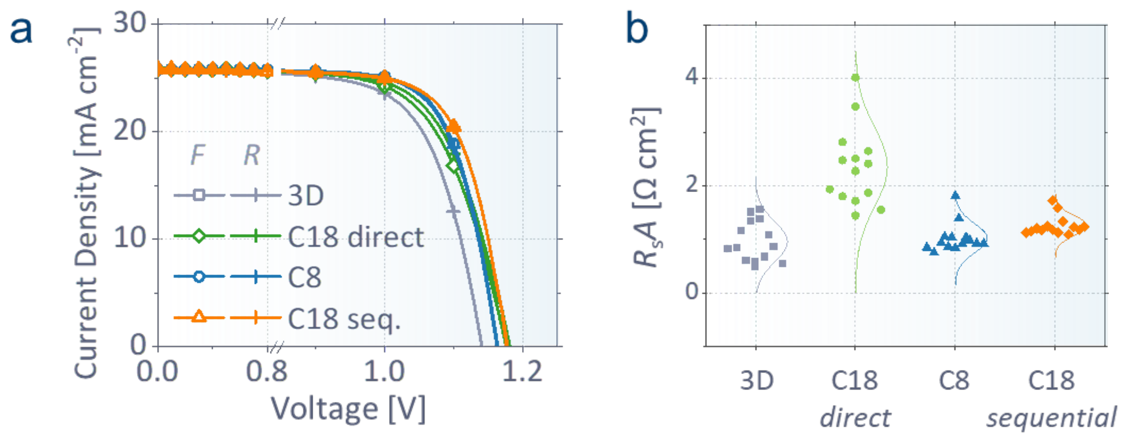

Supplementary Figure 17. a, Measured *J*–*V* curves and **b,** statistics of R_sA for the devices without and with various MQWs. The devices with direct coating of oleylamine (green) exhibit larger series resistances than those with OAI (blue) or sequential coating of OAI and oleylamine (orange).

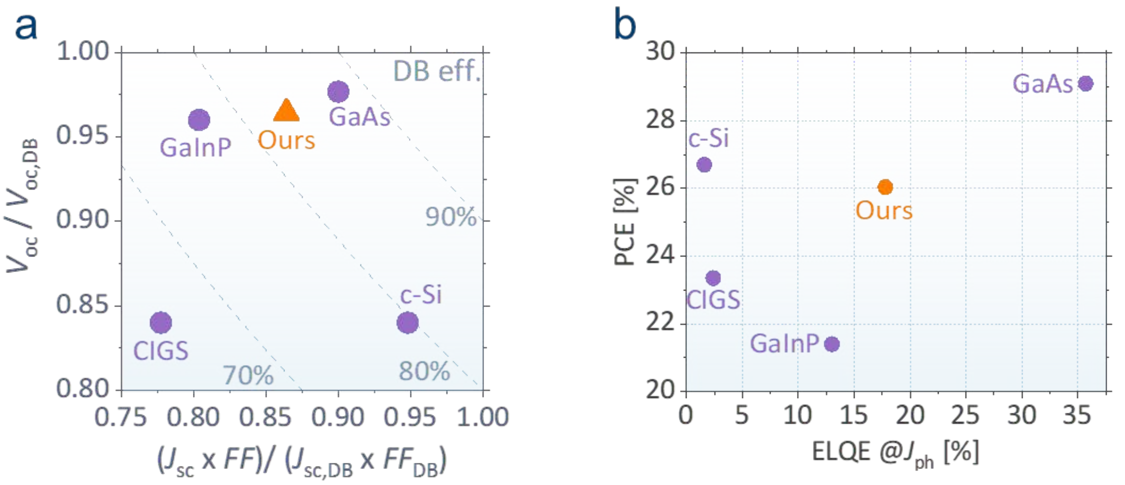

Supplementary Figure 18. a, Reported V_{oc} and $J_{sc} \times FF$ of various state-of-the-art photovoltaic devices and the proposed PPVs relative to their detailed balance limits for given bandgaps ($V_{oc,DB}$ and $J_{sc,DB} \times FF_{DB}$, respectively) [4,5]. **b,** Power conversion efficiency (PCE) and ELQE at J_{ph} of our PPVs compared to state-of-the-art photovoltaic devices based on GaAs, crystalline silicone (c-Si), copper indium gallium selenide (CIGS), and GaInP.

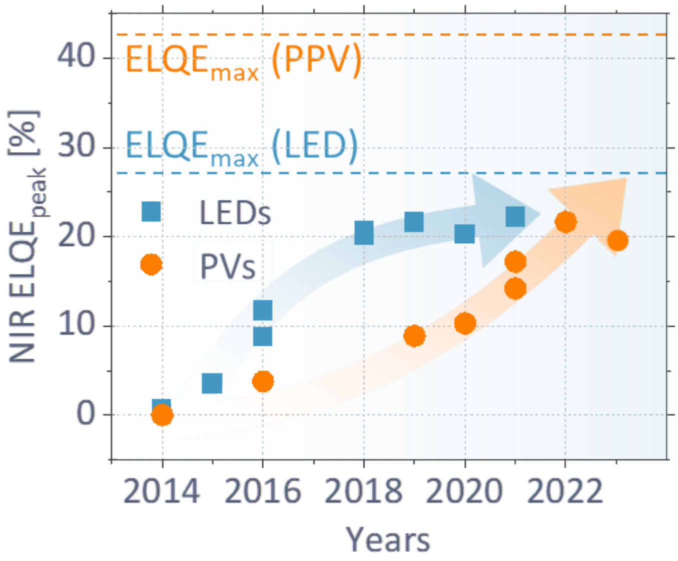

Supplementary Figure 19. Recent evolution of ELQEs of near-infrared-emitting perovskite optoelectronics reported as LEDs [6,7] or photovoltaic devices [3,8-13].

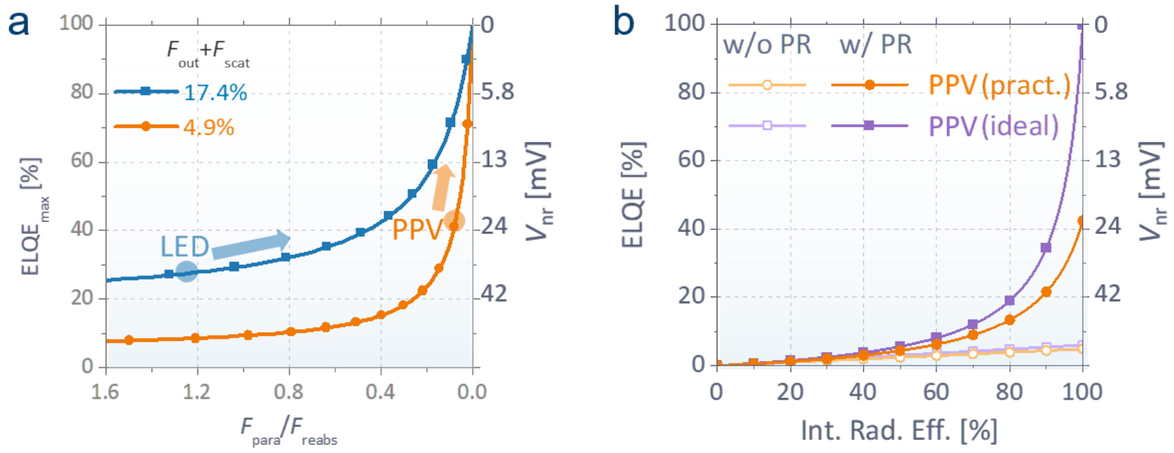

Supplementary Figure 20. a, Calculated maximum ELQEs and V_{nr} for various ratio of F_{para}/F_{reabs}, where $F_{out} + F_{scat}$ is fixed to 17.4% or 4.9% in equation 2. Two circled areas represent the properties of conventional architectures of LEDs and PPVs. **b,** Calculated ELQE and V_{nr} *versus* internal radiation efficiency (η_{rad}) for an ideal PPV structure replacing FTO with a completely non-absorbing material (n = 1.9) and Au with a perfect electric conductor. The maximum ELQE of 100% can be reached by such novel designs suppressing parasitic absorption and making $F_{out} + F_{scat} + F_{reabs}$ reach 100%.

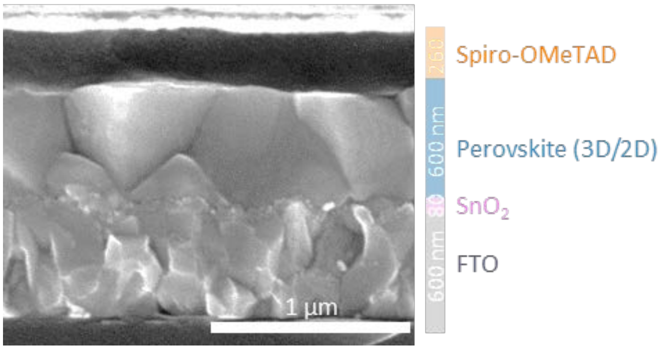

Supplementary Figure 21. Scanning electron microscope (SEM) image of the cross-section of our full device. The color bars represent the thicknesses we assumed for our simulations.

Supplementary References

1 Pazos-Outon, L. M. *et al.* Photon recycling in lead iodide perovskite solar cells. *Science* **351**, 1430-1433, doi:10.1126/science.aaf1168 (2016).

2 Cho, C. *et al.* The role of photon recycling in perovskite light-emitting diodes. *Nat. Commun.* **11**, 611, doi:10.1038/s41467-020-14401-1 (2020).

3 Cho, C. *et al.* Effects of photon recycling and scattering in high-performance perovskite solar cells. *Science Advances* **7**, eabj1363, doi:10.1126/sciadv.abj1363 (2021).

4 lmpv.amolf.nl/db.

5 Polman, A., Knight, M., Garnett Erik, C., Ehrler, B. & Sinke Wim, C. Photovoltaic materials: Present efficiencies and future challenges. *Science* **352**, aad4424, doi:10.1126/science.aad4424 (2016).

6 Kar, S., Jamaludin, N. F., Yantara, N., Mhaisalkar, S. G. & Leong, W. L. Recent advancements and perspectives on light management and high performance in perovskite light-emitting diodes. *Nanophotonics* **10**, 2103-2143, doi:doi:10.1515/nanoph-2021-0033 (2020).

7 Zhu, L. *et al.* Unveiling the additive-assisted oriented growth of perovskite crystallite for high performance light-emitting diodes. *Nat. Commun.* **12**, 5081, doi:10.1038/s41467-021-25407-8 (2021).

8 Saliba, M. *et al.* Incorporation of rubidium cations into perovskite solar cells improves photovoltaic performance. *Science* **354**, 206-209, doi:10.1126/science.aah5557 (2016).

9 Tvingstedt, K. *et al.* Radiative efficiency of lead iodide based perovskite solar cells. *Sci. Rep.* **4**, 6071, doi:10.1038/srep06071 (2014).

10 Yoo, J. J. *et al.* An interface stabilized perovskite solar cell with high stabilized efficiency and low voltage loss. *Energy Environ. Sci.* **12**, 2192-2199, doi:10.1039/C9EE00751B (2019).

11 Jeong, M. *et al.* Stable perovskite solar cells with efficiency exceeding 24.8% and 0.3-V voltage loss. *Science* **369**, 1615, doi:10.1126/science.abb7167 (2020).

12 Yoo, J. J. *et al.* Efficient perovskite solar cells via improved carrier management. *Nature* **590**, 587-593, doi:10.1038/s41586-021-03285-w (2021).

13 Zhao, Y. *et al.* Inactive (PbI2)2RbCl stabilizes perovskite films for efficient solar cells. *Science* **377**, 531-534, doi:10.1126/science.abp8873 (2022).
