# Supplementary material for: Quantum barriers engineering toward radiative and stable perovskite photovoltaic devices
Source: Nat Commun. 2024 May 28;15:4547. doi: 10.1038/s41467-024-48887-w (PMC11133308; doi:10.1038/s41467-024-48887-w)
Supplement: Supplementary file 3 — Reporting Summary [file 41467_2024_48887_MOESM3_ESM.pdf]

nature portfolio

Corresponding author(s): Neil C. Greenham and Jun Hong Noh

Solar Cells Reporting Summary

Nature Portfolio wishes to improve the reproducibility of the work that we publish. This form is intended for publication with all accepted papers reporting the characterization of photovoltaic devices and provides structure for consistency and transparency in reporting. Some list items might not apply to an individual manuscript, but all fields must be completed for clarity.

For further information on Nature Research policies, including our data availability policy, see Authors & Referees.

Experimental design

Please check the following details are reported in the manuscript, and provide a brief description or explanation where applicable.

1. Dimensions

Area of the tested solar cells
- [x] Yes — We used a mask with 0.0957 cm^2 (circle type).
- [ ] No — *Explain why this information is not reported/not relevant.*

Method used to determine the device area
- [x] Yes — The area of mask was determined by Newport (accredited certification center).
- [ ] No — *Explain why this information is not reported/not relevant.*

2. Current-voltage characterization

Current density-voltage (J-V) plots in both forward and backward direction
- [x] Yes — The J-V measured at Newport is presented in Supplementary Figure 12.
- [ ] No

Voltage scan conditions
- [x] Yes — Detailed conditions are described in the Method section.
- [ ] No — *Explain why this information is not reported/not relevant.*

Test environment
- [x] Yes — Our devices were evaluated in air without encapsulation at 25 degrees Celsius.
- [ ] No — *Explain why this information is not reported/not relevant.*

Protocol for preconditioning of the device before its characterization
- [x] Yes — To prevent reflection of incident light, an anti-reflection film was attached on glass side.
- [ ] No — *Explain why this information is not reported/not relevant.*

Stability of the J-V characteristic
- [x] Yes — Quasi-steady state (QSS) scans were performed by Newport. (Supplementary Figure 12, 13).
- [ ] No — *Explain why this information is not reported/not relevant.*

3. Hysteresis or any other unusual behaviour

Description of the unusual behaviour observed during the characterization
- [x] Yes — No J-V hysteresis was observed in the devices evaluated by Newport. (Supplementary Figure 12)
- [ ] No — *Explain why this information is not reported/not relevant.*

Related experimental data
- [x] Yes — Supplementary Figure 12a
- [ ] No — *Explain why this information is not reported/not relevant.*

4. Efficiency

External quantum efficiency (EQE) or incident photons to current efficiency (IPCE)
- [x] Yes — Supplementary Figure 11
- [ ] No — *Explain why this information is not reported/not relevant.*

A comparison between the integrated response under the standard reference spectrum and the response measure under the simulator
- [x] Yes — Comparisons can be found in Supplementary Figure 11 and Supplementary Figure 12.
- [ ] No — *Explain why this information is not reported/not relevant.*

For tandem solar cells, the bias illumination and bias voltage used for each subcell

☐ Yes
☐ No

Provide a description of the measurement conditions.

Explain why this information is not reported/not relevant.

5. Calibration

Light source and reference cell or sensor used for the characterization

☒ Yes
☐ No

We used a solar simulator(Class AAA-94043A, Newport) as the light source and a Si reference cell.

Explain why this information is not reported/not relevant.

Confirmation that the reference cell was calibrated and certified

☒ Yes
☐ No

We used a calibrated Si-reference cell certified by National Renewable Energy Laboratory (NREL).

Explain why this information is not reported/not relevant.

Calculation of spectral mismatch between the reference cell and the devices under test

☒ Yes
☐ No

Confirmed by certification at Newport. (Supplementary Figure 13)

Explain why this information is not reported/not relevant.

6. Mask/aperture

Size of the mask/aperture used during testing

☒ Yes
☐ No

We used a mask with 0.0957 cm^2 (circle type).

Explain why this information is not reported/not relevant.

Variation of the measured short-circuit current density with the mask/aperture area

☐ Yes
☒ No

Report the difference in the short-circuit current density values measured with the mask and aperture area.

The Jsc values for our devices were confirmed by Newport. (Supplementary Figure 12, 13)

7. Performance certification

Identity of the independent certification laboratory that confirmed the photovoltaic performance

☒ Yes
☐ No

Our devices are certified by Newport. (Supplementary Figure 12, 13)

Explain why this information is not reported/not relevant.

A copy of any certificate(s)

☒ Yes
☐ No

Supplementary Figure 12 and Supplementary Figure 13

Explain why this information is not reported/not relevant.

8. Statistics

Number of solar cells tested

☒ Yes
☐ No

15 devices were tested for statistical analysis of efficiency.

Explain why this information is not reported/not relevant.

Statistical analysis of the device performance

☒ Yes
☐ No

This is shown in Figure 3 and Supplementary Figure 12.

Explain why this information is not reported/not relevant.

9. Long-term stability analysis

Type of analysis, bias conditions and environmental conditions

☒ Yes
☐ No

Thermal stability and 2 year storage stability tests were performed in air without encapsulation. Continuous light stability tests were performed in air with encapsulation. (Figure 3)

Explain why this information is not reported/not relevant.
